# Phone Conversation while Processing Information: Chronometric Analysis of Load Effects in Everyday-media Multitasking

**DOI:** 10.3389/fpsyg.2017.00896

**Published:** 2017-06-06

**Authors:** Michael B. Steinborn, Lynn Huestegge

**Affiliations:** Psychologie III, Universität WürzburgWürzburg, Germany

**Keywords:** vigilance, sustained attention, cell phone conversation, variability, effort

## Abstract

This is a pilot study that examined the effect of cell-phone conversation on cognition using a continuous multitasking paradigm. Current theorizing argues that phone conversation affects behavior (e.g., driving) by interfering at a level of cognitive processes (not peripheral activity) and by implying an attentional-failure account. Within the framework of an intermittent spare–utilized capacity threading model, we examined the effect of aspects of (secondary-task) phone conversation on (primary-task) continuous arithmetic performance, asking whether phone use makes components of automatic and controlled information-processing (i.e., easy vs. hard mental arithmetic) run more slowly, or alternatively, makes processing run less reliably albeit with the same processing speed. The results can be summarized as follows: While neither expecting a text message nor expecting an impending phone call had any detrimental effects on performance, active phone conversation was clearly detrimental to primary-task performance. Crucially, the decrement imposed by secondary-task (conversation) was not due to a constant slowdown but is better be characterized by an occasional breakdown of information processing, which differentially affected automatic and controlled components of primary-task processing. In conclusion, these findings support the notion that phone conversation makes individuals not constantly slower but more vulnerable to commit attention failure, and in this way, hampers stability of (primary-task) information processing.

## Introduction

Everyday experience tells us that people have profound multitasking abilities since multitasking activities are extremely common in people’s everyday-life routines (cf. Bills, 1943, pp. 151–185; Salvucci and Taatgen, 2011, pp. 3–24). For example, researchers are often talking of running multiple projects concurrently, or are concurrently consuming multiple media sources at work and leisure. Sufficient practice provided, people might even be able to acquire superior everyday-life multitasking abilities (Ophir et al., 2009; Schubert et al., 2015), which is particularly true within the area of multimedia applications and gaming (Strobach et al., 2012). Not surprisingly, since the majority of actions and decisions is governed by routinized programs (Norman and Shallice, 1986; Langner and Eickhoff, 2013), the commonsense view of multitasking would lead one to expect that seemingly skilled behavior can concurrently be performed with ease, without any considerable impairment in performance (Finley et al., 2014). Thus, multitasking implies an advantage in time saving in the majority of standard situations, which often leads people to neglect the fact that it might also entail a disadvantage in unexpected situations, where rapid adaptations to changes are required (Hockey, 1997; Wickens, 2008; Parmentier, 2014). Taking up these issues, we focused on multitasking-induced performance (un)reliability, examining effects of phone usage on automatic and controlled components of information processing.

### Empirical Findings: Loading and Distraction Effects

Current empirical findings on continuous multitasking effects in the applied domain are largely dominated by two lines of research, by experiments on the effect of multitasking on learning and studying (Rohrer and Pashler, 2003; Pashler et al., 2013), and experiments on the effect of cell phone usage during driving (Alm and Nilsson, 1994; Horrey and Wickens, 2006). The experimental design in such applied studies is usually unconstrained, which brings about the benefits of retaining ecological validity (in some cases, at the cost of experimental control). Although there are (on principle) a variety of design options, the most typical experimental set-up found in the empirical literature usually consists of the following essential elements, a primary task which is usually performed in streams of continuous action, and a secondary task which is conceptualized either as a discrete event (e.g., an infrequent probe task) or a distractor such as a phone call (Sanders, 1998, pp. 271–285). The main line of empirical evidence stems from continuous tracking (or serial responding by key pressing) as the primary task and discrete manual or vocal responses to probe stimuli as the secondary task (Pashler, 1998, pp. 298–317). It is usually asked whether the loading/distractor affects primary-task performance, and the research question is mostly of practical relevance (Strayer and Johnston, 2001; Hancock et al., 2003; Strayer and Drews, 2007).

Empirically, there are three main determinants that affect primary task performance in natural contexts, in particular, the temporal predictability and task predictability of the secondary task, and controllability of the entire task ensemble (Sanders, 1998, pp. 330–359). These factors are ubiquitous and occasionally recognized as such (Kalsbeek and Sykes, 1967; Salvucci and Taatgen, 2008; Steinborn and Langner, 2011, 2012; Reissland and Manzey, 2016), albeit not strictly accounted for by the prevalent multiple-resource theory (Wickens, 1980, 1984). Specifically, when probe stimuli (as secondary task) occur at a constant rate within blocks of trials during the primary task, participants know exactly about when they will occur, and thus, are more likely to engage in appropriate processing strategies (Steinborn et al., 2016b). This again goes better when the nature of the secondary task is also constant, since task operations can better be prepared when these features are predictable as compared to when they are not (Kalsbeek and Sykes, 1967; Thomaschke and Dreisbach, 2015). Finally, multitasking depends greatly on whether individuals are enabled to do it their own way, that is, when they decided their own scheduling, than when they were to follow fixed schedules. For example, experiments reported by Hockey and Earle (2006) demonstrate that control over the regulation of multitask office work has an eminent impact on the way in which fatigue develops in response to demanding work goals (cf. Sanders, 1998, pp. 394–441).

Much of research in the applied domain is devoted to the effects of cell phone use during driving (e.g., Alm and Nilsson, 1994; Horrey and Wickens, 2006; Cooper and Strayer, 2008; Nijboer et al., 2016), and there is no doubt that this issue is of great practical importance and contributes much to a science-based approach to road safety policy (cf. Strayer and Drews, 2007). The essential finding can be summarized such that the use of mobile phones during driving leads to impairments at a purely cognitive (not peripheral) level thus increasing the risk of an accident. Strayer et al. (2003) examined the hypothesis of whether the observed impairment could be attributed to a disengagement of attention from the visual scene. Their results indicated that although an object is fixated, it is not being processed sufficiently (cf. Huestegge and Adam, 2011). In most countries, therefore, placing and receiving a phone call while driving are only allowed via hands-free systems. Yet, recent findings point to a reduction in attention directed toward the driving task even when using hands-free system car kits indicating that the source of interference produced by phone conversation originates not from manual operations related to phone use but from processing information related to conversation (Drews et al., 2008, 2009; Atchley et al., 2011a,b; Bergen et al., 2013).

Our theorizing given in the following section will finally converge toward an integrated spare–utilized capacity threading model as general framework (Kahneman, 1973). Two key aspects are of great importance. First, findings suggest that distraction effects by phone conversation are primarily caused through the cognitive effects of conversation and not (solely) by peripheral activities related to phone use. Second, the evidence delivers clues as to the possibility that phone-related distraction does not arise from a general slowing of relevant information-processing operations necessary for driving, but from an increase in the probability of attentional failure. For example, Casner and Schooler (2015, p. 38) examined vigilance-like phenomena in pilots performing routine tasks, concluding that people do not gradually become fatigued under vigilance conditions but occasionally jump into a rather discrete state of task-unrelated thoughts, or mind-wandering, respectively (Kurzban et al., 2013; Langner and Eickhoff, 2013; Smallwood, 2013; Steinborn et al., 2016b, for related accounts). Such a view of attention failure implies effects on performance variability which requires a theoretical model capable to explain the mechanism underlying performance fluctuations in active sustained-attention (i.e., mental-concentration) tasks (Pieters, 1983, 1985; Van Breukelen et al., 1995; Steinborn and Huestegge, 2016), and a spare–utilized capacity threading model offers a generic and integrated way of talking about performance variability.

### Theoretical Models: Spare–Utilized Capacity Threading

As already mentioned, we examined phone-related interference in active sustained-attention tasks to enable a chronometric approach to study applied-multitasking phenomena^1^. Central to any theorizing on performance speed and variability in active tasks is a distinction between utilized and spare capacity as structural and a continual swing between these processes as dynamic component, referred to as capacity threading. According to Kahneman (1973, 2013), the most generic way to theorize on the energetic regulation of capacity during continuous mental work is to consider information processing as composed of two qualitatively distinct and constantly alternating classes of mechanisms which he termed operating and monitoring (Craik, 1948; Welford, 1959). Both processes serve different purposes (within the same goal area) and are complementary to each other with regard to energetic requirements. This means that it is subjectively more demanding to engage in mental operations than to not engage (i.e., than to keep spare capacity available). A measure of spare capacity is obtained by analyzing the response to an infrequent probe signal, presented to the individual at an unpredictable time during the primary task (cf. Kahneman et al., 1967; Posner and Boies, 1971; Shulman and Greenberg, 1971). By means of this method, it is possible to determine the amount of (utilized) capacity that is deployed to the task at the instant of probe-signal presentation, and a failure to identify or an unusually slow response to the probe-signal indicates that the individual is currently absorbed in the effective mental operations of the task at hand.

Kahneman (1973) argued that as individuals actually engage in the mental operations of the task at hand, spare (fluctuating) capacity is conveyed to utilized capacity and the corresponding increase in task focus would lead to a (temporary) decrease in monitoring. For example, Kahneman et al. (1967) demonstrated that when people engage in highly demanding mental operations (in the add-1 task) for a short period of time (i.e., when they perform a cognitive sprint), they are virtually blind during that period as revealed by measures of the probe-signal technique. In this way, he considered capacity allocation for an impending task as mobilization of mental energy (recruited from available spare capacity) to enable active mental operations. Mobilization is transient and time-sensitive, which means that it is virtually impossible to voluntarily sustain attention for more than a few seconds within one continuous stream of mental work. From this perspective, sustained attention is considered a mere re-implementation of successive efforts to redirect attention (to retransform spare to utilized capacity) to the task at hand. Thus, even when individuals have the intention to deliberately concentrate on the task for a while, capacity will never fully be utilized at any point during that period, but there is always spare capacity left for monitoring, evaluating, and adjusting pre-set performance standards (cf. Steinborn et al., 2016b).

Such a perspective of spare–utilized capacity threading offers a very natural way to explain variability in active mental tasks where response time (RT) is the primary performance measure. Instead of attributing experimental effects on RT variability to unspecified or umbrella-like terms often used in the literature (e.g., mental noise, ego depletion, lack of motivation, etc.), the model provides a generic and clearly defined mechanism based on spare–utilized capacity threading, according to which variations occur because the allocation policy sometimes channels capacity to other activities, resulting in slower responses during that period of trials (Steinborn et al., 2016b). This directly implies that the RT distribution of an individual is composed of a mixture between two operating mental states, an attentive state and a non-attentive state (cf. Luce, 1986, pp. 273–311; Ulrich and Miller, 1994, pp. 34–36; Van Breukelen et al., 1995, pp. 150–169). In the attentive state, the individual is effectively carrying out mental operations while in non-attentive periods, the individual is not effectively working because utilized capacity is conveyed to spare capacity. Note that this view has some decidedly important properties to explain performance fluctuations beyond mere scaling-variability (Wagenmakers and Brown, 2007), as indicated by relativized indices such as the RT coefficient of variation (RTCV), which is obtained by dividing the intraindividual RT standard deviation by the mean (cf. Steinborn et al., 2016b).

Although the advantage of RT variability and distributional analysis is widely recognized in the basic-research domains, researchers and practitioners in applied-research domains still rely on traditional measures of central tendency. A chronometric approach to studying performance speed and its fluctuation strictly implies a methodology beyond measures of central tendency, which can be studied in a comfortable way by analyzing the cumulative distributive function (CDF) of responses. The reason is that effects on RT mean are not interpretable by itself if they originate from a selective slowing at longer CDF percentiles (Miller, 2006). RT distributions are typically asymmetrical, having a steep slope on the left side (due to a rather narrow range of very fast responses) but an elongated right tail (arising from more broadly distributed slow responses)^2^. Thus, RT variability expresses itself chiefly in responses above RT mean, and many variables affect RT mean only indirectly by selectively affecting stability (cf. Steinborn and Huestegge, 2016; Steinborn et al., 2016b). In the foreground of a research project within a spare–utilized capacity threading model thereby stand the goals of manipulating effort mobilization directly and measuring its effects with high precision by analyzing the entire RT distribution instead of only analyzing RT means (Steinborn et al., 2016a, 2017). This might provide a principal advancement to previous studies in this domain (cf. Sanders, 1998, pp. 394–451).

### Present Study

Most work on driver distraction by cell phone conversation focused on the assessment of the impairment rather than on a delineation of the cognitive mechanisms underlying deficits in driving performance. Yet, studies that focused on this question imply an attention-failure account rather than a constant slowing of information-processing activity. In the present study, we aimed to precisely estimate potential impairments of cognitive performance by everyday-life cell phone usage, particularly by talking and texting. Our study can be characterized by two key aspects: First, we used an unconstrained continuous-multitasking paradigm. This is commonly accepted in applied-research domains, however, our approach differs in some way to previous studies since we used self-paced mental arithmetic as primary task, examining performance alone and in combination with unconstrained cell-phone conversation as secondary-task. We decided to use continuous arithmetic in order to enable the application of chronometric methods of RT measurement (Manzey and Lorenz, 1998; Haque and Washington, 2014). Further, we used a naturalistic conversation as secondary task, according to the methodical suggestions of Drews et al. (2008, pp. 393–395), to retain maximal ecological validity (Amado and Ulupinar, 2005; Horrey and Wickens, 2006). Second, in the foreground of our research thereby stands the use of advanced performance-measurement methodology to critically capture aspects of performance reliability.

Notably though, the bulk of current research on cell-phone distraction neglected this important aspect of measurement. Whether the hypothesized effects on performance originate from a constant slowing of the speed of information processing, or alternatively, by an increase in the probability of attention failure is fundamental to the analysis and understanding of cell-phone distraction. In order to distinguish between both theoretical alternatives, we need to go beyond traditional measures of central tendency but instead must consider its effect at critical density zones of the entire RT distribution (Balota and Spieler, 1999; Spieler et al., 2000). We computed a CDF for each experimental condition, asking whether phone-related impairments during continuous cognitive processing makes information processing run more slowly, or alternatively, makes processing run less reliably albeit with the same processing speed. Notably, this distinction is critically implied by current theorizing, albeit not explicitly measured in driving tasks (Groeger, 1999). Consequently, we examined whether experimental effects on RT mean originate from a global slow-down that is equally present at all CDF percentiles (parallel effect) or only from a local effect at slower percentiles (mixture effect). The former would indicate a true influence of continuous information-processing speed while the latter would indicate a destabilization of performance (Steinborn et al., 2016a, 2017).

Globally, we expected to observe an effect of cell-phone conversation on measures of RT and accuracy. That is, responses should be faster and somewhat less erroneous under the single-task condition as compared to a multi-task condition (main effect of context). We further expected faster responses for easy mental arithmetic as compared to hard mental arithmetic (main effect of demand). Whether cell-phone conversation differentially affects easy versus hard mental arithmetic performance is an empirical question, since previous research on continuous multitasking does not deliver enough reliable information on the impact of conversation on automatic versus controlled information processing components (cf. Ashcraft and Battaglia, 1978; Logan, 1979; Borst et al., 2013). To examine behavioral variability, we analyzed both the classic parameters of RT variability and parameters of distributional skewness based on the ex-Gaussian model (cf. Heathcote et al., 1991; Leth-Steensen et al., 2000). Remind that from the perspective of an energetic spare–utilized capacity threading model, it is crucial to know whether cell-phone conversation during cognitive processing leads to a generic (vs. selective) slow-down of all (vs. only long) CDF percentiles. Theorizing within an energetic-capacity framework, conversation is expected to hamper information-processing by increasing the probability of attentional failure. We examined both the effect of expecting and performing phone talking (Experiment 1) and text communication (Experiment 2) on cognitive performance, using the same sample of participants (within the sequence of both experiments counterbalanced across participants).

## Materials and Methods

### Participants

A student-based sample of 39 (29 female, 10 male) volunteers (mean age = 23.5 years, *SD* = 6.5) took part in the experiment. All participants were in standard condition (reported to be healthy) and had normal or corrected-to-normal vision.

### Apparatus and Stimuli

The experiment was programmed using PsychoPy (Peirce, 2009). Participants sat about 60 cm in front of the screen. To mimic the characteristic (i.e., self-regulated) features of active continuous information-processing, we used mental arithmetic as one of the primary cultural techniques (Thorndike, 1922; Bills, 1943), practiced among identifiable cultural groups, and amenable to advanced psychometric analysis. In particular, we used a version of the mental-addition and verification task that contained both easy and hard items, using a short response–stimulus interval of 50 ms, which is particularly suitable to examine performance fluctuations (Sanders and Hoogenboom, 1970; Soetens et al., 1985; Steinborn et al., 2010, 2012). In each trial, an addition term together with the result is presented and participants indicated whether the result is either correct or incorrect. They were instructed to verify a correct result by pressing the right key (right index finger) and to falsify an incorrect result by pressing the left key (left index finger). The task contained easy and difficult items differing with respect to the chain length. Items categorized as easy included simple additions (e.g., 4 + 5 = 9; 4 + 5 = 8) while items categorized as difficult included chained additions (e.g., 4 + 5 + 1 + 2 = 12; 4 + 5 + 1 + 2 = 11). There were 24 easy items and 24 hard items. Each item was presented randomly and equally often (total of 865 trials).

### Automatic and Controlled Processing Components

In the present study, we used easy (chain length = 1) and hard (chain length = 4–5) mental-addition items as a proxy for automatic versus controlled processing components in mental arithmetic, which is well-agreed and theoretically backed-up by exemplar-based theories of cognition, learning, and automaticity. This consequently leads to a distinction between two general modes of solving mental-addition problems, a calculation-based mode and one that is based on memory retrieval. In the human-factors domain, this is often referred to as workload (albeit in a more intuitive way) and in most cases, not further specified. For example, Logan (1988) considered performance as automatic when it is based on single-step, direct-access retrieval of solutions from memory, while he considered performance as controlled when it is based on algorithmic processing mechanisms such as counting, addition, memorizing, or borrowing (Groen and Parkman, 1972; Ashcraft, 1992; Imbo et al., 2007). It should be clear that the use of this terminology only makes sense when the context in which the terminology is employed, is also specified (Logan, 1988, pp. 493–495). Crucial is the assumption that every encounter of a stimulus (e.g., 4 + 5 = 9) results in episodic recording and retrieval, given the individual is sufficiently attentive and responsive. More formally, this leads to a set of fundamental assumptions: Attending deliberately to an event such as a single mental-arithmetic problem furnishes obligatory encoding and obligatory retrieval of separate instances in memory. Stimulus processing is characterized in terms of a race between algorithmic processing and memory retrieval such that whichever finishes first in a particular trial controls the response. In other words, any mental-arithmetic problem in a particular trial is finally solved either by the former or the latter process.

### Procedure

For practical reasons, we decided to examine both the effect of texting (Experiment 1) and of phone talking (Experiment 2) on cognition, using the same sample of participants, with these experimental blocks counterbalanced across participants. Each experiment contained a single-task condition (mental arithmetic was performed alone), an expected-load condition (participants anticipated an interruption by an incoming text message or phone call, respectively), and a performed-load condition (mental arithmetic was performed in combination with a memory load or active talking, respectively). Crucially, the text message (Experiment 1) was presented prior to task processing (in order to measure expectancy unconfounded with real task processing), and the expected-load condition (phone call, Experiment 2) occurred shortly after the experimental block. Notably, due to the difficulty to randomize the expected-load condition, we decided to present the three critical experimental conditions in a fixed order (single task, expected load, performed load), which means that differences between the conditions are confounded with potential task order effects. This has important consequences as research hypotheses can only be tested in one direction. That is, we are allowed to ask questions about expected and performed dual-task interference but not about potential benefits (and consequently, the same applies to the interpretation of potential effects). We averaged the single-task condition in order to cushion the impact of all kinds of test-taker effects (i.e., fatigue, practice effects, etc.). Apart from that, one half of the sample was first administered with Experiment 1 (texting: single, expected, and performed) followed by Experiment 2 (phone talking: single, expected, and performed), and the other half of the sample was administered in the counterbalanced order. They were introduced with the experimental paradigm and were instructed to concentrate throughout the experimental session, that is, to respond with maximum speed and accuracy (cf. Ulrich and Miller, 1994).

### Implementation of Phone-Call Expectancy (Experiment 1)

Whether the sole expectation to receive a call from a student colleague affects cognitive performance is an empirical question, and although most people would agree with such a hypothesis from everyday experience, it is difficult to experimentally manipulate aspects of pure expectation such that it mimics the naturalistic aspects of phone calls in real life, regarding relevance and time pressure to answer the impending phone call. Therefore, the nature of this aspect of our study is exploratory, and only serves to obtain a first impression from the detailed analysis of cognitive processes derived from automatic and controlled processing components of continuous mental addition. The procedure was such that the experimenter informed the participant that he/she will get a phone call during the processing of the task and instructed the participant to answer the call as quickly as possible. The phone lied in front of the participant on the table and called out to him/her to be picked up and used.

### Implementation of Phone Conversation (Experiment 1)

We used the method of story-based natural conversation (cf. Drews et al., 2008), using a scripted semi-structured interview guideline, to mimic the coordinated, joint-activity features of a naturalistic everyday-life small talk conversation among students. The screenplay resembled the method of improvisational theater and contained the following essential elements in the following order (1) become acquainted with each other, telling names, etc. (2) asking about how he/she is doing today, (3) asking about where he/she is living, in which part of the city, etc., (4) asking about what he/she had for lunch, etc., (5) asking about what courses are offered this semester, which courses he/she is currently attending, which his/her favorite lecture and/or professor is, (6) and, for example, what could define a possible motto for the “psychoparty” (an annual party arranged by third-semester students). Further, the interview contained optional elements to ensure the conversation to flow appropriately in the eventual case of the participant being either extremely talkative or taciturn (i.e., to regulate turn-taking). Our aim hereby was to establish a conversation with balanced conversation’s proportion, regulating each partner’s contribution to the talk toward a value of about 50% (maximal tolerable deviation about 40–60 or 60–40%. These questions were also chosen among a sample of standard questions to retain smalltalk, for example, asking whether he/she like animals, whether he/she is doing sports, whether he/she likes hot wetter, whether he/she knows certain proverbial sayings, etc.

### Implementation of Text Message Expectancy

In a similar way, another exploratory aspect of our study contained the question of whether the sole expectation to receive a text message from a student colleague in some way affects cognition. For example, everyday experience would imply that expecting a text message from a student colleague during a lecture (where the phone is not allowed to be used actively) potentially distracts individuals such that attention is directed away from the content of the lecture toward the potential content of the message. The procedure was such that the experimenter informed the participant that he/she will get text a message during the processing of the task and instructed the participant not to answer the message until the experimental block is finished. Again, the phone lied before the participant on the table and called out to him/her to be picked up and used.

### Implementation of Text Message Communication

The participant obtained a text message and was asked the following questions: “Was wäre ein optimales Geburtstagsgeschenk für Dich, wenn der Preis egal wäre?” (“What would you want as a birthday present? What would you choose, if money is no object?”). The participant was instructed to think about an answer during the processing of the task and to answer this questions after finishing the experimental block.

## Results (Experiment 1: Texting)

### Data Treatment

Responses faster than 100 ms were regarded outliers and removed from RT analysis. To effectively take advantage of the full scope of distributional analysis, we only used a minimal-trimming method by removing the three slowest reactions for each of the conditions, according to Ulrich and Miller (1994), and in accordance with our previous use of this method (e.g., Steinborn and Huestegge, 2016). Incorrect responses were regarded response errors and used to compute an index of error rate.

### Standard Performance Indices

For each of the experimental conditions, we computed the reaction time mean (RTM) to index average response speed and the RTCV to index relative response-speed variability, according to the suggestion of Flehmig et al. (2007) and Flehmig et al. (2010), and according to our previous use of this method. RTCV is obtained by computing the standard deviation of the RTs (separately for each individual and experimental condition) divided by the individual mean of RTs (for each individual and experimental conditions). Error percentage (EP) indicated the rate of incorrect responses, and served as measure of response accuracy.

### Distributional Analysis

To analyze the distribution of responses, we computed the interpolated vincentized CDF of responses with 19 percentiles for each of the experimental conditions according to the suggestion of Ulrich et al. (2007). By means of this analysis, we were to know whether the hypothesized effect of phone conversation is due to a generic slow-down of all responses or alternatively due to a selective slow-down of the long percentiles of the CDF. To more directly account for experimentally induced effects of distributional shape (right-tail density accumulation effects, further referred to as skewness), we additionally adopted an ex-Gaussian model approach but only as a descriptive model of reaction times to analyzing its three parameters mean, dispersion, and shape (μ, σ, and τ). We computed ex-Gaussian model parameters for each participant according to the methodical rules provided by Lacouture and Cousineau (2008). Within the context of a chained mental-arithmetic task, parameters μ and σ can readily be interpreted as localization and dispersion (around μ) indicators while τ is sensitive to experimental effects on right-tail density accumulation (Steinborn and Huestegge, 2016).

### Standard Analysis

**FIGURE 1:**
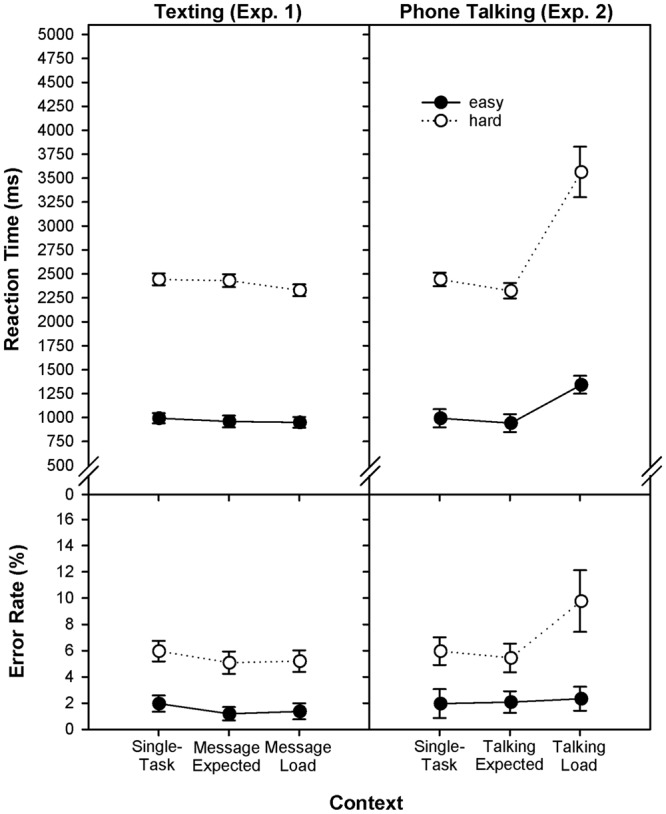
Reaction time mean and error rate (RTM, EP) as a function of the factors context (single-task vs. expected load vs. load) and demand (easy vs. hard) in continuous mental arithmetic, separately displayed for Experiments 1 (texting) and 2 (phone talking).

The design contained the experimental factors context (single task vs. expecting text message vs. handling text message) and demand (easy vs. hard mental arithmetic) and contained RT and error rate as dependent measures. Complete statistical results are referred to in **Table 1** and visually displayed in **Figure 1**. Responses became not slower in the experimental blocks as compared to the single-task condition. As expected, responses were faster in easy than in hard mental-arithmetic trials, as indicated by a main effect of demand on RTM [*F*(1,38) = 653.7, *p* < 0.01]. Finally, multitasking did not differentially impose negative effects on automatic and controlled components of mental arithmetic. Finally, it should be mentioned that there was no speed-accuracy trade-off that could compromise the interpretation of effects on RT. In fact, errors were low overall and therefore not further considered (cf. Steinborn et al., 2017).

**Table 1:** Mean reaction time (RT) and standard error of the mean (SE) as a function of the factors context and demand, separately for Experiments 1 and 2 (texting vs. talking).

	Factor levels		Experiment 1				Experiment 2			
	Context	Demand	RT (ms)		EP (%)		RT (ms)		EP (%)	
			*M*	*SE*	*M*	*SE*	*M*	*SE*	*M*	*SE*
1	1	1	993	31.6 (26.6)	1.96	0.23 (0.32)	992	31.55 (47.98)	1.96	0.23 (0.56)
2	1	2	2444	73.6 (31.5)	5.96	0.61 (0.40)	2444	73.58 (36.14)	5.96	0.61 (0.54)
3	2	1	959	28.2 (31.1)	1.19	0.28 (0.26)	941	27.21 (47.72)	2.08	0.43 (0.42)
4	2	2	2431	78.5 (34.7)	5.08	0.68 (0.44)	2324	79.11 (41.54)	5.44	0.68 (0.55)
5	3	1	950	30.7 (29.0)	1.37	0.24 (0.31)	1343	63.04 (47.42)	2.32	0.86 (0.47)
6	3	2	2330	72.2 (31.3)	5.20	0.62 (0.42)	3565	181.48 (134.59)	9.76	1.70 (1.20)

*N = 39; RT, reaction time mean; EP, error rate (%); *M* and *SE* are population parameters, and SE is transformed for within-subject designs according to Cousineau (2005), shown in brackets.*

### Distributional Analysis

**FIGURE 2:**
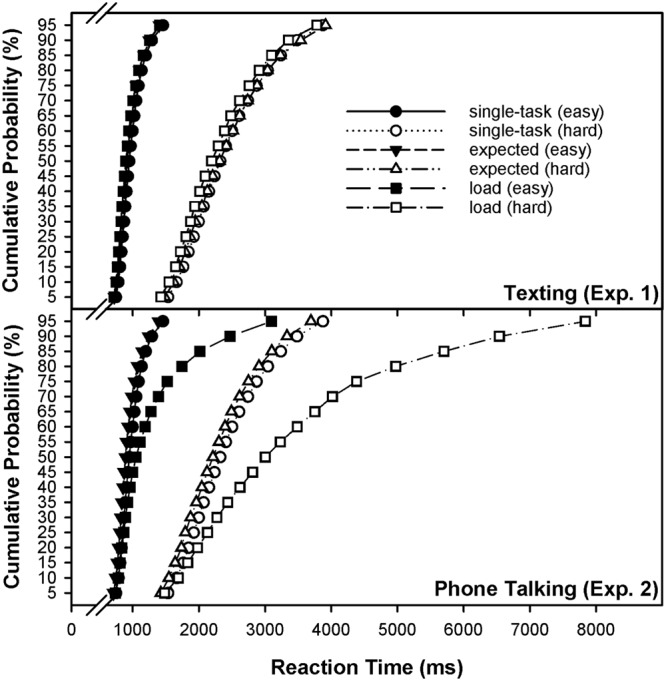
Vincentized and interpolated cumulative distributive function (CDF) of reaction times for each combination of the factors context (single-task vs. expected load vs. load) and demand (easy vs. hard) in continuous mental arithmetic, separately displayed for Experiments 1 (texting) and 2 (phone talking).

Besides effects on average response speed, we hypothesized that multitasking increased performance variability in the primary task, which should perhaps be more pronounced for hard than for easy mental arithmetic. However, a visual inspection of the CDFs (**Figure 2**) indicates that neither expected nor performed text message communication severely affected aspects of distributional skewness in the mental-arithmetic task. There was no significant effect on any parameter of performance variability, with respect to the global GLM effect (**Tables 1–3**).

**Table 2:** Results of the experimental effects on standard performance indices (Experiment 1).

			RTM			EP			RTCV		
	Source: Overall	df	*F*	*p*	*η^2^*	*F*	*p*	*η^2^*	*F*	*p*	*η^2^*
1	Context	2,76	11.1	0.000	0.23	3.5	0.039	0.08	2.9	0.073	0.07
2	Demand	1,38	653.7	0.000	0.95	61.6	0.000	0.62	112.0	0.000	0.75
3	Context × Demand	2,76	6.0	0.004	0.14	0.0	0.960	0.00	1.1	0.326	0.03
	**Source: Single vs. Expected**										
4	Context	1,38	1.6	0.211	0.04	8.2	0.007	0.18	1.8	0.186	0.05
5	Demand	1,38	653.7	0.000	0.95	61.6	0.000	0.62	112.0	0.000	0.75
6	Context × Demand	1,38	0.5	0.474	0.01	0.0	0.856	0.00	1.0	0.319	0.03
	**Source: Single vs. Load**										
7	Context	1,38	17.6	0.000	0.32	3.9	0.055	0.09	5.4	0.026	0.12
8	Demand	1,38	653.7	0.000	0.95	61.6	0.000	0.62	112.0	0.000	0.75
9	Context × Demand	1,38	6.4	0.015	0.15	0.1	0.801	0.00	2.2	0.144	0.06

**Table 3:** Results of the experimental effects on ex-Gaussian parameters (Experiment 1).

			μ (Mean)			σ (Variability)			τ (Skewness)		
	Source: Overall	df	*F*	*p*	*η^2^*	*F*	*p*	*η^2^*	*F*	*p*	*η^2^*
1	Context	2,76	18.8	0.000	0.33	2.9	0.074	0.07	2.6	0.085	0.06
2	Demand	1,38	331.0	0.000	0.90	97.3	0.000	0.72	164.0	0.000	0.82
3	Context × Demand	2,76	7.9	0.001	0.17	1.6	0.220	0.04	2.7	0.074	0.07
	**Source: Single vs. Expected**										
4	Context	1,38	5.1	0.030	0.12	0.3	0.566	0.01	2.1	0.159	0.05
5	Demand	1,38	331.0	0.000	0.90	97.3	0.000	0.72	164.0	0.000	0.82
6	Context × Demand	1,38	1.8	0.118	0.05	0.4	0.523	0.01	3.3	0.077	0.08
	**Source: Single vs. Load**										
7	Context	1,38	49.1	0.000	0.56	9.7	0.003	0.20	5.9	0.020	0.14
8	Demand	1,38	331.0	0.000	0.90	97.3	0.000	0.72	164.0	0.000	0.82
9	Context × Demand	1,38	20.4	0.000	0.35	4.9	0.032	0.12	6.0	0.019	0.14

## Results (Experiment 2: Phone Talking)

### Standard Analysis

The design contained the experimental factors context (single task vs. expecting phone call vs. phone talking) and demand (easy vs. hard mental arithmetic) and contained RT and error rate as dependent measures. Complete statistical results are referred to in **Tables 3, 4**. Essentially, responses were significantly faster in single-task blocks as compared to the experimental condition, indicating that multitasking affects RTM [*F*(2,76) = 79.1, *p* < 0.01]. As expected, responses were faster in easy than in hard mental-arithmetic trials, as indicated by a main effect of demand on RTM [*F*(1,38) = 422.0, *p* < 0.01]. More interesting, multitasking differentially affected automatic and controlled components of mental arithmetic, since hard items were more affected than easy items [*F*(2,76) = 28.6, *p* < 0.01]. Pre-planned single-comparison analyses revealed that the global GLM effect is driven by the talking-load condition (single task vs. phone talking), indicating a slowing of responses which was differentially more pronounced for hard than for easy arithmetic (**Table 3**: Panels 7–9). Finally, it should be mentioned that there was no speed-accuracy trade-off that could compromise the interpretation of effects on RT. Errors were in the same direction, thus supporting the conclusion that talking load differentially hampers primary-task processing.

**Table 4:** Results of the experimental effects on standard performance indices (Experiment 2).

			RTM			EP			RTCV		
	Source: Overall	df	*F*	*p*	*η^2^*	*F*	*p*	*η^2^*	*F*	*p*	*η^2^*
1	Context	2,76	79.1	0.000	0.68	4.0	0.047	0.10	220.9	0.000	0.85
2	Demand	1,38	422.0	0.000	0.92	62.4	0.000	0.62	23.7	0.000	0.47
3	Context × Demand	2,76	28.6	0.000	0.43	8.9	0.001	0.19	6.1	0.003	0.14
	**Source: Single vs. Expected**										
4	Context	1,38	10.3	0.003	0.21	0.3	0.583	0.01	2.9	0.097	0.07
5	Demand	1,38	422.0	0.000	0.92	62.4	0.000	0.62	23.7	0.000	0.47
6	Context × Demand	1,38	3.7	0.063	0.09	0.7	0.402	0.02	0.0	0.882	0.00
	**Source: Single vs. Load**										
7	Context	1,38	70.3	0.000	0.65	3.4	0.073	0.08	247.0	0.000	0.87
8	Demand	1,38	422.0	0.000	0.92	62.4	0.000	0.62	23.7	0.000	0.47
9	Context × Demand	1,38	26.2	0.000	0.41	10.8	0.002	0.22	7.9	0.008	0.17

### Distributional Analysis

Besides effects on average response speed, multitasking increased the performance variability of the primary task. Notably, a visual inspection of the CDFs (**Figure 2**) indicates that multitasking affected primary-task performance by destabilizing performance. This is indicated by a main effect of context on the classic variability parameter, RTCV [*F*(2,76) = 220.9, *p* < 0.01], which closely corresponds to the visual pattern of skewness of a particular CDF. The main effect of the factor demand on RTCV indicates greater variability for hard than for easy items [*F*(1,38) = 23.7, *p* < 0.01], and the context × demand interaction on RTCV indicates that multitasking evoked performance variability to a larger degree in the controlled than in the automatic component of mental arithmetic [*F*(2,76) = 6.1, *p* < 0.01]. Since recommended by several authors (Heathcote et al., 1991; Steinhauser and Huebner, 2009), we additionally obtained parameter of skewness from an ex-Gaussian distributional model. As expected, the destabilizing effect on performance is also (even more sensitively) indicated by a main effect of the factor context on the ex-Gaussian τ parameter [*F*(2,76) = 97.0, *p* < 0.01], and by the context × demand interaction effect on the τ parameter [*F*(2,76) = 36.0, *p* < 0.01]. Pre-planned single-comparison analyses revealed that the global GLM effect is driven by the talking-load condition (single task vs. phone talking), indicating a slowing of responses which was differentially more pronounced for hard than for easy arithmetic (**Tables 4, 5**: Panels 7–9). Thus, results indicate that phone talking during continuous primary-task performance affects not simply the speed of information-processing throughput (Humphreys and Revelle, 1984; Thorne, 2006; Steinborn et al., 2010), but crucially, the reliability of these processes, supporting an attentional-failure hypothesis rather than a general slow-down hypothesis of smartphone-conversation effects on cognitive work.

**Table 5:** Results of the experimental effects on ex-Gaussian parameters (Experiment 2).

			μ (Mean)			σ (Variability)			τ (Skewness)		
	Source: Overall	df	*F*	*p*	*η^2^*	*F*	*p*	*η^2^*	*F*	*p*	*η^2^*
1	Context	2,76	7.9	0.002	0.17	2.0	0.150	0.07	97.0	0.000	0.72
2	Demand	1,38	434.0	0.000	0.92	98.3	0.000	0.72	138.6	0.000	0.77
3	Context × Demand	2,76	3.3	0.040	0.08	0.6	0.527	0.04	36.0	0.000	0.49
	**Source: Single vs. Expected**										
4	Context	1,38	9.1	0.005	0.19	0.8	0.353	0.02	0.1	0.706	0.00
5	Demand	1,38	434.0	0.000	0.92	98.3	0.000	0.72	138.6	0.000	0.77
6	Context × Demand	1,38	3.1	0.087	0.08	0.5	0.484	0.01	0.3	0.576	0.01
	**Source: Single vs. Load**										
7	Context	1,38	14.0	0.001	0.27	3.9	0.054	0.09	107.4	0.000	0.74
8	Demand	1,38	434.0	0.000	0.92	98.3	0.000	0.72	138.6	0.000	0.77
9	Context × Demand	1,38	6.7	0.014	0.15	1.2	0.290	0.03	43.7	0.000	0.54

## Discussion

### Summary

The aim of this applied study was to examine the effects of (secondary-task) smartphone communication on (primary-task) performance in chained mental arithmetic. The results can be summarized as follows: (1) Contrary to popular opinion, neither the sole expectation of an impending text message (a question) nor the mental preoccupation with finding an answer (to the question delivered by the text message) had any detrimental effect on primary-task performance. (2) Further, the expectation of an impending phone call was also not detrimental to primary-task performance. (3) However, active conversation was clearly detrimental to primary-task performance, since responses were slower on average in the talking-load condition as compared to the single-task condition. (4) Importantly, talking did not yield a constant slowing but rather a destabilization of continuous mental-arithmetic performance, since the CDF analysis revealed increased distributional skewness beyond scaling variability. (5) The destabilization effect was more pronounced for hard than for easy items, indicating a differential effect on controlled versus automatic components of mental arithmetic. This result might be important to our understanding of how smartphone communication affects cognitive functioning in general, and might also be of applied importance because it may help to understand better how phone conversation impacts on a driver’s ability to allocate attention to the task of driving.

### Effects of Expected Multimedia-Based Communication

Most people would agree, when asked, that impending but temporally uncertain social interaction at the workplace or elsewhere is distracting and can sometimes be even annoying. Further, researchers and practitioners in applied fields would also agree that multimedia-based communication devices represent the biggest distraction at work, despite the methodical difficulties to develop a model (i.e., a micro-case) that exactly mimics the interactive features of multimedia-based communication in natural environments (Ralph et al., 2014, 2015). Therefore, the results presented here are of explorative character, although they might reveal aspects that are relevant for the practical use in future studies. Contrary to our expectations, and to popular beliefs based on everyday experience, expecting an impending text message (containing a question) in our study did not hamper performance in the primary task (Experiment 1). Further, the load imposed with finding an answer (to the question delivered by the text message) did also not detrimentally affect any aspect of primary-task performance (**Figures 1, 2**). However, it would be premature to conclude that impending text messages are unproblematic with regard to possible distraction effects on primary-task performance, since one cannot definitely exclude that more demanding text messages might affect performance in the primary task.

In Experiment 2, we asked whether the expectation to receive a call from a student colleague affects cognitive performance in the primary task. Likewise as in Experiment 1, expecting an impending phone call was not at all detrimental to primary-task performance. In contrast, actively performed conversation (talking) was clearly detrimental to primary-task performance, since responses were slower on average in the talking-load condition as compared to the single-task condition. Thus, these data would indicate the conclusion that impending phone-call expectancy is not harmful to the individual currently engaged in deliberate information-processing activity, which is counterintuitive to what one would expect from everyday experience. Such findings are often interpreted such that the anticipation of impending distraction could have evocated additional capacity (or enforced a strategy of cognitive shielding) and by this means prevented any impairment of primary-task performance to occur (Fuentes and Campoy, 2008; Bratzke et al., 2009, 2012; Langner et al., 2010, 2011; Szalma and Hancock, 2011; Scheiter et al., 2014). Due to the chosen design features of our study, however, hypotheses could only be formulated in one direction (i.e., toward potential dual-task interference costs, not benefits), and results will thus only be interpreted accordingly. Instead, some critical issues are outlined below.

Critical to a manipulation of expected-load effects (Experiment 1) are two aspects, **(1)** the nature and degree of demand related to processing a text message, **(2)** and the experimental means of performing controls to determine secondary-task engagement (i.e., to determine how long and how intensely the participants were processing the text message). Critical to a manipulation of expectancy for an impending phone call (Experiment 2) are two further aspects, **(3)** the experimental methods of inducing an internal state of hurry (i.e., the problem of getting the participants to act with the required urgency), **(4)** and the methods of controlling for when exactly and how often the participants re-started to preparing for the anticipated event. Within a spare–utilized capacity threading model and related accounts, these intrusions are reflected in aspects of intraindividual performance variability. For example, McDaniel et al. (2004) considered several aspects relevant to study performance costs related to a monitored event. As a general rule, it is important to ascertain whether the phone call can easily be detected perceptually (e.g., phone nearby in sight vs. far-apart, ringing loud vs. muted, etc.), whether the occurrence uncertainty is event-based or time-based (e.g., phone call expected after lunch, or at around 12.00 am), and whether there is time pressure to answer the call pointing on a distinction between immediate-execute vs. delayed-execute secondary-task mode (McDaniel and Einstein, 2000; McDaniel et al., 2004; Einstein and McDaniel, 2005).

### Effects of Phone Conversation (Talking)

Researchers usually agree with the allegation that active conversation requires attention for monitoring semantic aspects such as topic and content, for coordinating the time-critical aspect of turn taking (between speaking and listening), and for a rather metacognitive supervision of conversational balance (Kahneman, 1973, pp. 5–12; Salvucci and Taatgen, 2011, pp. 3–24). This means that conversation is a complex matter affected by many factors and therefore difficult to examine in a wholistic fashion (Drews et al., 2008; Bergen et al., 2013). Notably, the problem is actually recognized and is a current point of contention among researchers in basic-research and applied-research domains (cf. Drews et al., 2008, pp. 393–395). In the present study, we decided to examine the effect of phone conversation on cognition by means of the classic continuous dual-task paradigm, where a primary task is performed in streams of continuous action, and where a secondary task is used as a loading or distractor condition, or to “probe” mental focus during primary-task processing (Posner and Boies, 1971, pp. 401–407). Our results indicate that active phone conversation had an enormous impact on mental-arithmetic performance, since the load imposed by conversation yielded slower and somewhat more erroneous responses, as compared to a standard (single-task) condition (**Figure 1**).

**FIGURE 3:**
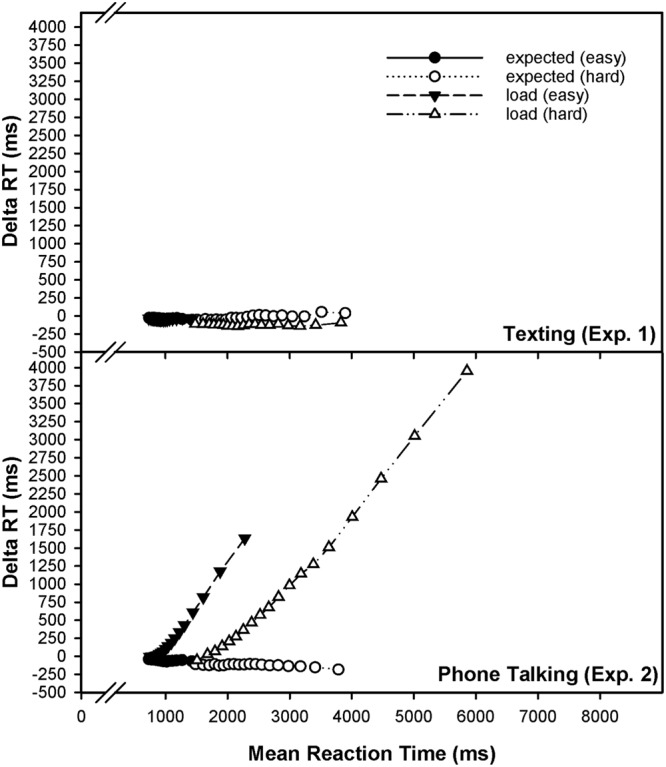
Delta plots of the distraction effect by cell-phone usage. For each percentile, the RT difference between the experimental conditions (single-task vs. expected load; single-task vs. load) is plotted against the mean of the conditions in that percentile. Data are separately displayed for Experiments 1 (texting) and 2 (phone talking).

Importantly, phone talking not solely slowed but rather destabilized primary-task performance, as indicated by measures of response-speed variability, which was again more pronounced for hard than for easy mental arithmetic (**Tables 4, 5**). Thus, the decrements on RT mean are not interpretable by itself (cf. Miller, 2006, p. 93), since it could be demonstrated that they actually originate from a selective slow-down of responses at long CDF percentiles. It becomes evident from **Figure 2** that the experimental conditions (standard vs. talking load) are not very different at the shorter percentiles of the CDF while the difference increases substantially toward the longest percentiles. **Figure 3** displays a delta plot of the loading effect on mental-arithmetic performance, comparably for the low-demand and the high-demand condition. A delta plot is obtained by calculating the RT difference as induced by an experimental manipulation (e.g., single-task vs. load) against the mean of both experimental conditions for each of the percentiles. By this means, effects of concurrent phone conversation can be evaluated relative to the mean of level of performance, indicating that individuals were not particularly going slower overall but especially became less persistent. In this way, delta plots provide a convenient simplification of the relatively complex information present in the CDFs (cf. De Jong et al., 1994; Ridderinkhof, 2002; Schwarz and Miller, 2012; Ulrich et al., 2015; Steinborn et al., 2016b).

Therefore, the main conclusion our study provides is that phone conversation during mental arithmetic does not globally hamper information-processing speed. Rather, the data indicate that load of this kind makes individuals less reliable and less capable to protecting the cognitive system against attention failure. The probability of committing such failures of attention depends on the processing demand of the primary task, being lower for easy than for hard mental arithmetic. This indicates that secondary-task conversation differentially affects automatic and controlled information processing in the primary task. In this way, our study might contribute some important aspects to the understanding of phone-conversation effects on everyday-life tasks such as driving, despite the fact that we employed a continuous mental-arithmetic task to study conversation-related attentional impairments. Thus, particular key characteristics of our study might be those of creating connections between basic and applied research along the concept of a two-state model of attentional failures. For example, Briem and Hedman (1995) concluded that simple phone conversation is in itself not sufficient to adversely affect the ability to maintain road position, but rather increases the risk of traffic accidents by an unfortunate coincidence of a critical traffic event and spontaneous attention failure within the individual (cf. Reason, 1990; Folkard, 1997).

### Theory and Design Issues

Our theorizing is primarily based upon an intermittent spare–utilized capacity threading model as a general framework, in order to account for two essential findings. The first relates to the empirical fact that loading effects by phone conversation on primary-task performance can primarily be located at a cognitive (not at a peripheral-activity) level. The second refers to the possibility, implied by previous findings, that phone-related interference does impose a constant amount of costs (of sharing capacity) on primary-task performance, but temporarily blocks information processing in the primary task (by a processing bottleneck) in an all-or-none fashion. In this way, our study diverges from the majority of applied multitasking research where the theorizing usually occurs within the multiple-resource model framework. For example, Wickens (1984) originally assumed that successful multitasking depends on the compatibility of input systems, representational format, and output systems. From this account, one would have to argue that (auditory–verbal–vocal) phone conversation may be performed concurrently with little or no costs to a (visual–spatial–manual) task as continuous mental arithmetic. Given the apparent three-dimensional compatibility of this dual-task combination, phone conversation and continuous mental arithmetic should be performed together with no interference, which was obviously not the case in our study (cf. Strayer and Drews, 2007).

The basic tenet of a spare–utilized capacity threading (monitoring–focus) model is that there is an intermittent exchange between capacity for task operations and for monitoring. Crucial is the notion of intermittency, as task processing is interrupted during monitoring, which means that as individuals engage in active task operations, spare capacity is conveyed to utilized capacity. Thus, a temporary increase in task focus would yield a corresponding (temporary) decrease in monitoring. A spare–utilized capacity threading model is not only consistent but even relies on the notion of a processing bottleneck, as it assumes that individuals can (effectively) engage in only one of the two, task processing or monitoring. In this way, it is mutually exclusive with the notion of (temporarily–punctual) sharing of capacity. This means that the relation of utilized versus spare capacity is constantly fluctuating across subsequent trials as this relation is continually evaluated and re-adjusted, which means that capacity for active task operations varies across trials. Remind that several empirical findings of Strayer et al. (2003) support this position, suggesting that even when talking drivers direct their gaze at objects in the environment, they often fail to see them. To put it more precisely, there is an increased probability for drivers currently engaged in active conversation to commit attention failure (in a temporarily punctual fashion) to recognize objects in the environment.

A theoretical alternative to the prevalent multiple-resource framework in applied-multitasking research (Wickens, 1980, 1984), therefore, is that conversation-related interference stems from an intermittent postponement imposed by a discrete-processing bottleneck such that attending to the phone conversation temporarily blocks information processing in the primary task, because the bottleneck forces serial processing between talking and performing continuous arithmetic. Such a view of trial-by-trial intermittent resource allocation offers a completely natural way to explain variability that is usually observed in RT experiments. In (low-error domain) RT tasks, these trial-by-trial fluctuations in the rate of utilized capacity (task focus) are reflected in the right tail (skewness) of the intraindividual RT distribution. The results of the present study are completely in line with such a perspective: As visually displayed in **Figures 2, 3**, individuals were partially capable to retain a high level of primary-task performance during talking (as compared to the standard condition), which is particularly true for automatic (vs. controlled) processing, but are partially prone to commit a failure to engage in processing the primary task. That is, they are not very different at the shortest percentiles of the CDF while the difference increases substantially toward the longest percentiles, and this effect differentially depends on the demand imposed by the primary task. According to Strayer and Drews (2007), conversation is special in that thought packages cannot be broken into arbitrary units but instead is composed of turns that engage the central-processing bottleneck.

### Final Conclusion

A final word ought be devoted to the fundamental question of how an applied multitasking study should be conducted in order to satisfy the requirements of ensuring experimental control (i.e., internal validity), on the one hand, and to provide representativeness of the created micro case (i.e., external validity), on the other hand. Although the problem has been recognized by theoreticians of outstanding reputation (e.g., Pashler, 1998, pp. 5–31; Sanders, 1998, pp. 452–506; Salvucci and Taatgen, 2011, pp. 237–253), no definite solution has been offered probably because the problem is unsolvable as it is a problem of perspective. An essential characteristic of applied research relates to the complexity of the real-life situation and the variety of possible influences and effect mechanisms. The dilemma is that isolating the separate influence of the independent variables increases internal validity but decreases representativeness. For example, Drews et al. (2008) criticized the frequently observed practice of reducing complexity to increase experimental control to study conversation-related interference, arguing that many tasks employed to simulate conversation in studies on cell phone use on driving suffer from serious ecological-validity concerns. For example, several studies used “verbal tasks” as representative for conversation, administering participants to decide between words and non-words, or to perform verbal-reasoning tasks as secondary-task assumed to interfering with primary-task performance. In any case, artificial tasks fail to mimic the features of real conversation.

We used the method of story-based natural conversation, using a scripted interview guideline, to simulate the self-regulated dyadic-activity characteristics of naturalistic everyday small talk conversation among students. The interview was semi-structured but contained optional elements to ensure the conversation to flow appropriately. We intended to establish a talking-load condition with balanced conversation’s proportion, being aware that varying the proportion between listening and speaking might be a potential source of interference measurable in continuous dual-task situations. For example, McCarley et al. (2004) found impairments in the ability of participants to detect changes in real-world traffic scenes when they were conversing on a hands-free device, however, no such performance decrements were observed when participants listened to prerecorded conversations from other participants. These findings are important since they demonstrate that listening to verbal material is by itself not sufficient to produce the dual-task interference associated with using a cell phone while driving. In any case, a more in-depth analysis of the particular components of real-life conversation is vital for the complete understanding of conversation-related interference in future studies. For the time being, we conclude that phone-related interference effects on cognition does not arise from a constant slow down but from an occasional break down of mental efficiency during continuous mental arithmetic performance.

The key contribution of our study embraces two aspects, knowledge related to the particular component processes affected by conversation in continuous dual-task situations, methodology of design and experimental set-up (Steinborn et al., 2017), and advanced measurement technology (Steinborn et al., 2016b). *First*, our results provide knowledge to the community since we determined component processes related to automatic and controlled information processing as they were affected by concurrent phone conversation. *Second*, we provide a methodical advancement to study conversation-related interference on primary-task performance within the framework of mental chronometry. In the focus of our research project stands the goal of measuring the effects of phone talking on automatic and controlled information processing with high precision, by analyzing the entire RT distribution instead of only analyzing RT means. The main conclusion our study provides is that interference by phone conversation is not due to a constant slowdown but rather due to an occasional breakdown of continuous information processing, which differentially affects automatic and controlled components of information processing. In effect, we argue that phone conversation makes individuals vulnerable to attention failure (being greater for controlled vs. automatic components), and in this way, hampers stability of information-processing throughput (Humphreys and Revelle, 1984; Steinborn et al., 2016b).

## Ethics Statement

Written informed consent was obtained from the participants regarding their agreement with their participation in this research. Our study was in accordance with the ethical standards of the institutional and national research committee and with the 1964 Helsinki declaration and its later amendments or comparable ethical standards.

## Author Contributions

Ideas and theorizing: MS and LH. Experiment and data analysis: MS. Interpreting results: MS and LH. Writing of the manuscript: MS.

## Conflict of Interest Statement

The authors declare that the research was conducted in the absence of any commercial or financial relationships that could be construed as a potential conflict of interest.

## Footnotes

1 In response to a reviewer’s comment, we would like to note that it is important to distinguish between active and more passive (i.e., vigilance-like) sustained-attention tasks when theorizing within a spare–utilized capacity threading model (cf. Langner and Eickhoff, 2013). To effectively engage in mental operations (e.g., such as counting, mental addition, or subtraction), the individual has to utilize capacity from available spare capacity, which is termed energetic mobilization (cf. Sanders, 1998, pp. 332–348). In contrast, in rather passive watchkeeping tasks (also referred to as vigilance tests, monitoring task, etc.), the individual’s primary task is to wait and watch for relevant targets, and the factor demand is typically increased by lowering target occurrence probability and by increasing negative consequences of missing the target (cf. Broadbent, 1971, pp. 76–111). In this situation, there is no threading between operating and monitoring but a conflict between task-related target monitoring and task-unrelated (mind-wandering) tendencies, which are extremely difficult to resolve. The difficulty originates from a basic attentional principle, namely that attention primarily serves action and is to be attained and maintained through acting (cf. Neumann and Prinz, 1987). According to Kahneman (1973, pp. 13–27), it is virtually impossible to mobilize capacity in wait-and-watch tasks because they do not require action for most of the time (see also, Casner and Schooler, 2015, for a similar view).

2 Response to a reviewer’s comment. As mentioned earlier, the responses of an individual in RT tasks are not symmetrically distributed around the mean but are typically skewed such that they have an elongated tail toward the right. This distributional asymmetry is due to the fact that there is fundamental limit to maximizing response speed but none to response slowing. For example, the classic work of Bills (1931, 1935) devoted particular attention to the occurrence of incidental extra-long responses (which he termed “mental blocks”) after periods of normal work speed in self-paced color naming. Crucially, it is not a matter of the scale properties (i.e., being finite toward the left but infinite toward the right), as occasionally stated in the literature, but because of a limitation in the speed of processing even when performed with maximum mental efficiency (Steinborn et al., 2016b). To illustrate this, consider a formula-one driver on a particular training day where everything clicks into place (e.g., driver is fully concentrated, check processes occur at exactly the critical moments, team coordination is effective, etc.). On this day, the hypothetical lap times of the driver will be almost always near to ideal line (e.g., 73, 71, 71, 74, 76, 73, 74 s, etc.). Now consider a day where everything is not going as well as it should (e.g., driver not concentrated, team coordination ineffective, etc.). On this day, the driver may likely succeed in some (even in the majority of the) laps but may fail in other ones due to the particular circumstances on this particular day (e.g., 72, 71, 85, 74, 75, 93, 73 s, etc.). Critically, inspecting only measures of central tendency would lead to the conclusion that the driver was simply slower on the bad (vs. the good) day, which is convenient albeit incorrect (or at least incomplete) given that the overall slowing originated from an unfortunate combination of circumstances in some but not all of the rounds yielding extraordinarily slow lap times.
